# 690. Analysis of high-risk antibiotics for Clostridioides difficile infections in Korean hospitals: A nationwide multi-center study

**DOI:** 10.1093/ofid/ofad500.752

**Published:** 2023-11-27

**Authors:** Bongyoung Kim, Rangmi Myung, Jieun Kim, Uh Jin Kim, Yeon-Sook Kim, Eu Suk Kim, Tark Kim, Dae Won Park, Mi Suk Lee, Sun Hee Lee, Chang-Seop Lee, Hyun-Ha Chang, Myoung-jae Lee, Hyunjoo Pai

**Affiliations:** Department of Internal Medicine, Hanyang University College of Medicine, Seongdong-gu, Seoul-t'ukpyolsi, Republic of Korea; National Health Insurance Service, Wonju, Kangwon-do, Republic of Korea; Hanyang university hospital, Guri, Kyonggi-do, Republic of Korea; Chonnam National University Medical School, GwangJu, Kwangju-jikhalsi, Republic of Korea; Division of Infectious Diseases, Department of Internal Medicine, Chungnam National University School of Medicine, Daejeon, Taejon-jikhalsi, Republic of Korea; Seoul National University Bundang Hospital, Bundang-gu, Kyonggi-do, Republic of Korea; Department of Internal Medicine, Soonchunhyang University Bucheon Hospital, Bucheon, Kyonggi-do, Republic of Korea; Korea University Ansan Hospital, Ansansi, Kyonggi-do, Republic of Korea; Division of Infectious Diseases, Department of Internal Medicine, Kyung Hee University School of Medicine, Seoul, Seoul-t'ukpyolsi, Republic of Korea; Division of Infectious Disease, Department of Internal Medicine, Pusan National University Hospital, Seo-gu, Pusan-jikhalsi, Republic of Korea; Department of Internal Medicine, Jeonbuk National University Medical School, Jeonju, Cholla-bukto, Republic of Korea; Division of Infectious Diseases, Department of Internal Medicine, School of Medicine, Kyungpook National University, Daegu, Korea, Daegu, Taegu-jikhalsi, Republic of Korea; Department of Economics, College of Political Science and Economics, Korea University, Seoul, Seoul-t'ukpyolsi, Republic of Korea; Department of Internal Medicine, Hanyang University College of Medicine, Seongdong-gu, Seoul-t'ukpyolsi, Republic of Korea

## Abstract

**Background:**

Clostridioides difficile infection (CDI) is a representative healthcare-associated infection, and the incidence rate continues to increase in Korea. The purpose of this study is to identify antibiotics that are highly related to CDI incidence in Korean hospitals.

**Methods:**

From January-December 2019, all antibiotic prescription records, CDI test results, and a daily number of hospitalized patients were retrospectively collected from 10 university-affiliated hospitals in Korea. To exclude duplication, CDI tests that were conducted within 10 days after conducting previous CDI tests were excluded. When a positive result was detected from C. difficile toxin assay or PCR tests, we considered it as CDI. Antibiotics were defined as drugs corresponding to J01 in the WHO ATC classification, excluding anti-tuberculosis drugs, anti-parasitic drugs, antiviral drugs, and local antibiotics. The antibiotic usage was calculated as Days of Therapy (DOT). The CDI incidence rate and antibiotic usage were collected on a weekly basis and then corrected to 1,000 patient days. The correlation between CDI incidence rate and antibiotic usage was analyzed using the cross-correlation function test.

**Results:**

CDI incidence and total antibiotic usage in each hospital ranged from 0.49-1.58/1,000 patient-days and 588.7-1000.4 DOT/1,000 patient-days, respectively. Cross-correlation function test shows that antibiotics that are highly related to CDI incidence were 2nd generation cephalosporin that was prescribed 6 weeks prior to CDI event (coefficient 0.320, P = 0.021), 4th generation cephalosporin that was prescribed 1 week prior to CDI event (coefficient 0.307, P = 0.027), and beta-lactam/beta-lactamase inhibitors (with anti-pseudomonal effects) that was prescribed 1 week prior to CDI event (coefficient 0.272, P = 0.050), lincosamide that was prescribed 2 weeks prior to CDI event (coefficient 0.278, P = 0.045), and oxazolidinone that was prescribed 8 weeks prior to CDI event (coefficient 0.362, P = 0.008).

Cross-correlation coefficients between Clostridioides difficile infection incidence and antibiotic usage

**Figure:**
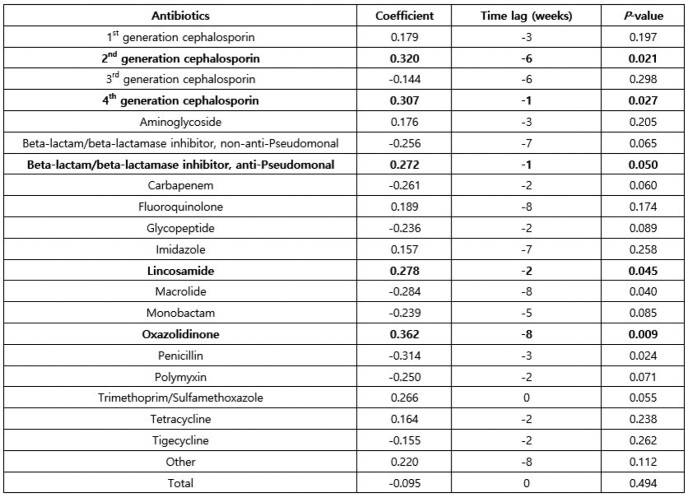

**Conclusion:**

Antibiotics highly associated with CDI incidence in Korean hospitals were second-generation cephalosporin, fourth-generation cephalosporin, beta-lactam/beta-lactamase inhibitors (with anti-pseudomonal effects), lincosamide, and oxazolidinone.

**Disclosures:**

**All Authors**: No reported disclosures

